# Supplementary material for: Thalamic regulation of frontal interactions in human cognitive flexibility
Source: PLoS Comput Biol. 2022 Sep 12;18(9):e1010500. doi: 10.1371/journal.pcbi.1010500 (PMC9499289; doi:10.1371/journal.pcbi.1010500)
Supplement: S1 Table — (DOCX) [file pcbi.1010500.s001.docx]

**S1 Table.** **Brain regions related to the Strategy Switching (*Switching* > *Staying*) (p < 0.001, uncorrected)**

| Regions | Hemisphere | Peak coordinates | | | T-score |
| --- | --- | --- | --- | --- | --- |
|  |  | x | y | z |  |
| Insula | R | 44 | 20 | -4 | 6.89 |
| Insula | L | -30 | 24 | 4 | 9.45 |
| **Dorsolateral prefrontal cortex** | **R** | **46** | **30** | **34** | **7.07** |
| Dorsolateral prefrontal cortex | L | -38 | 30 | 36 | 7.16 |
| Premotor area | R | 34 | 2 | 62 | 7.41 |
| Premotor area | L | -26 | 0 | 62 | 8.03 |
| **Orbitofrontal cortex** | **R** | **28** | **56** | **-6** | **7.31** |
| Orbitofrontal cortex | L | -34 | 52 | 0 | 6.43 |
| Supplementary motor cortex | L&R | 0 | 22 | 46 | 9.40 |
| Inferior parietal lobule | R | 46 | -44 | 56 | 7.95 |
| Inferior parietal lobule | L | -32 | -50 | 42 | 9.27 |
| Precuneus | R | 4 | -62 | 62 | 5.84 |
| Precuneus | L | -10 | -66 | 50 | 7.12 |
| Caudate | L | -10 | 0 | 10 | 6.71 |
| **Mediodorsal thalamus** | **R** | **12** | **-10** | **8** | **4.37** |
| Brain stem | R | 2 | -26 | -24 | 3.42 |

Regions shown in bold indicate the regions of interest in the study (right dlPFC, right OFC and right MD)
