# Supplementary material for: Thalamic regulation of frontal interactions in human cognitive flexibility
Source: PLoS Comput Biol. 2022 Sep 12;18(9):e1010500. doi: 10.1371/journal.pcbi.1010500 (PMC9499289; doi:10.1371/journal.pcbi.1010500)
Supplement: S2 Table — (p < 0.001, uncorrected). (DOCX) [file pcbi.1010500.s002.docx]

**S2 Table**. **Brain regions correlating with the prior belief in *Switching* and *Staying.* (p < 0.001, uncorrected)**

| Regions | Hemisphere | Peak coordinates | | | T-score | |
| --- | --- | --- | --- | --- | --- | --- |
|  |  | x | y | z | |  |
| ***Switching*** | | | | | | |
| **Ventromedial prefrontal cortex** | **L&R** | **4** | **56** | **-8** | | **4.93** |
| Posterior cingulate cortex | L&R | 6 | -48 | 24 | | 5.30 |
| Middle cingulate cortex | L&R | 0 | -10 | 36 | | 4.98 |
| Precentral cortex | R | 40 | -12 | 50 | | 4.01 |
| Middle temporal cortex | L | -60 | -54 | 8 | | 4.35 |
| ***Staying*** | | | | | | |
| **Ventromedial prefrontal cortex** | **L&R** | **-6** | **62** | **2** | | **7.9** |
| Precuneus | L&R | -8 | -52 | 22 | | 6.89 |
| Middle cingulate cortex | L&R | 4 | -12 | 40 | | 6.02 |
| Middle temporal gyrus | L | -40 | -66 | 22 | | 6.77 |
| ParaHippocampus | R | 38 | -28 | -10 | | 5.75 |
| ParaHippocampus | L | -28 | -40 | -12 | | 6.15 |
| Postcentral cortex | R | 58 | -2 | 14 | | 5.72 |
| Postcentral cortex | L | -58 | -6 | 12 | | 5.03 |
| Precentral cortex | R | 22 | -26 | 68 | | 5.35 |
