# Supplementary figures and images for: Thalamic regulation of frontal interactions in human cognitive flexibility
Source: PLoS Comput Biol. 2022 Sep 12;18(9):e1010500. doi: 10.1371/journal.pcbi.1010500 (PMC9499289; doi:10.1371/journal.pcbi.1010500)

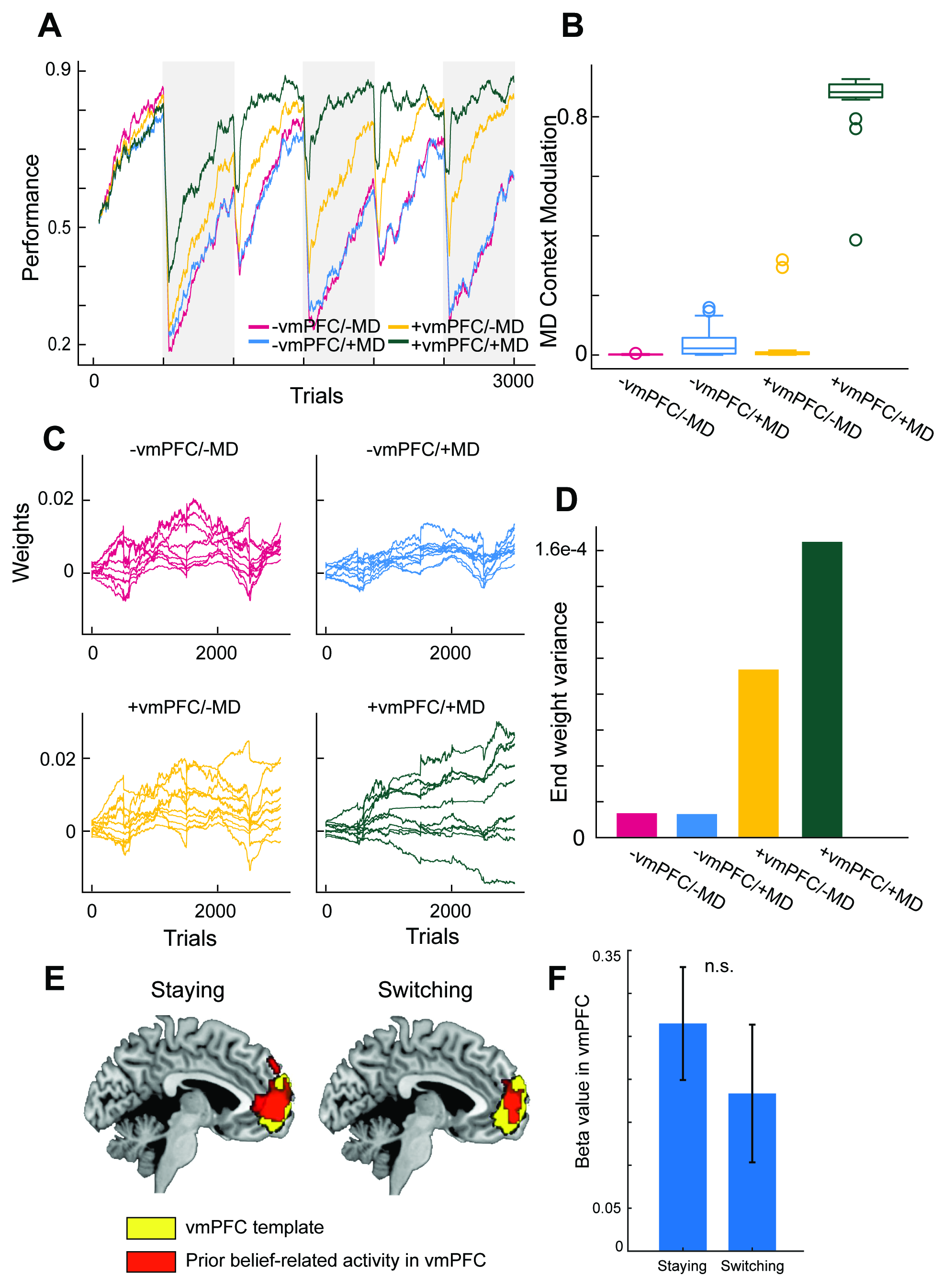

Supplement: S1 Fig — We compared the model with and without value input (+vmPFC, -vmPFC respectively), and also with and without output from MD (+MD, -MD, respectively). A. Performance of model with no value inputs shows little behavioral flexibility with significant dips in performance at block changepoints. B. Trial-averaged MD activity correlation with ground truth present context for each trial. Only model of both value inputs and intact MD output to dlPFC showed appropriate encoding of context in MD. C. The weights from dlPFC neurons to output showed significant learning and unlearning in model without value input. Adding value inputs leads to more coherent weight changes across blocks, and adding MD further reduce destructive learning and unlearning. D. We considered the distribution of output weights at the end of experiment and looked at its variance as a measure of dispersion. Weights that learned and then unlearned across blocks remained close to zero with low variance. E. The comparison of prior belief–related fMRI activity in vmPFC between Switching and Staying in human participants. Prior belief about the outcome value, derived from Hierarchical Gaussian Filter model (details in previously published papers [32,37]), correlated with the activity in vmPFC for both Staying and Switching strategy. The results projected on axial MRI brain slices. Brain activations displayed at p < 0.001 (uncorrected, red). For other regions see S2 Table. F. The prior belief-related activity (beta value) in vmPFC extracted from both Staying and Switching were applied to a paired sample t-test. The result showed no significant difference between Staying and Switching (p = 0.30). The error bar depicts the standard error. (TIF) [file pcbi.1010500.s003.tif]

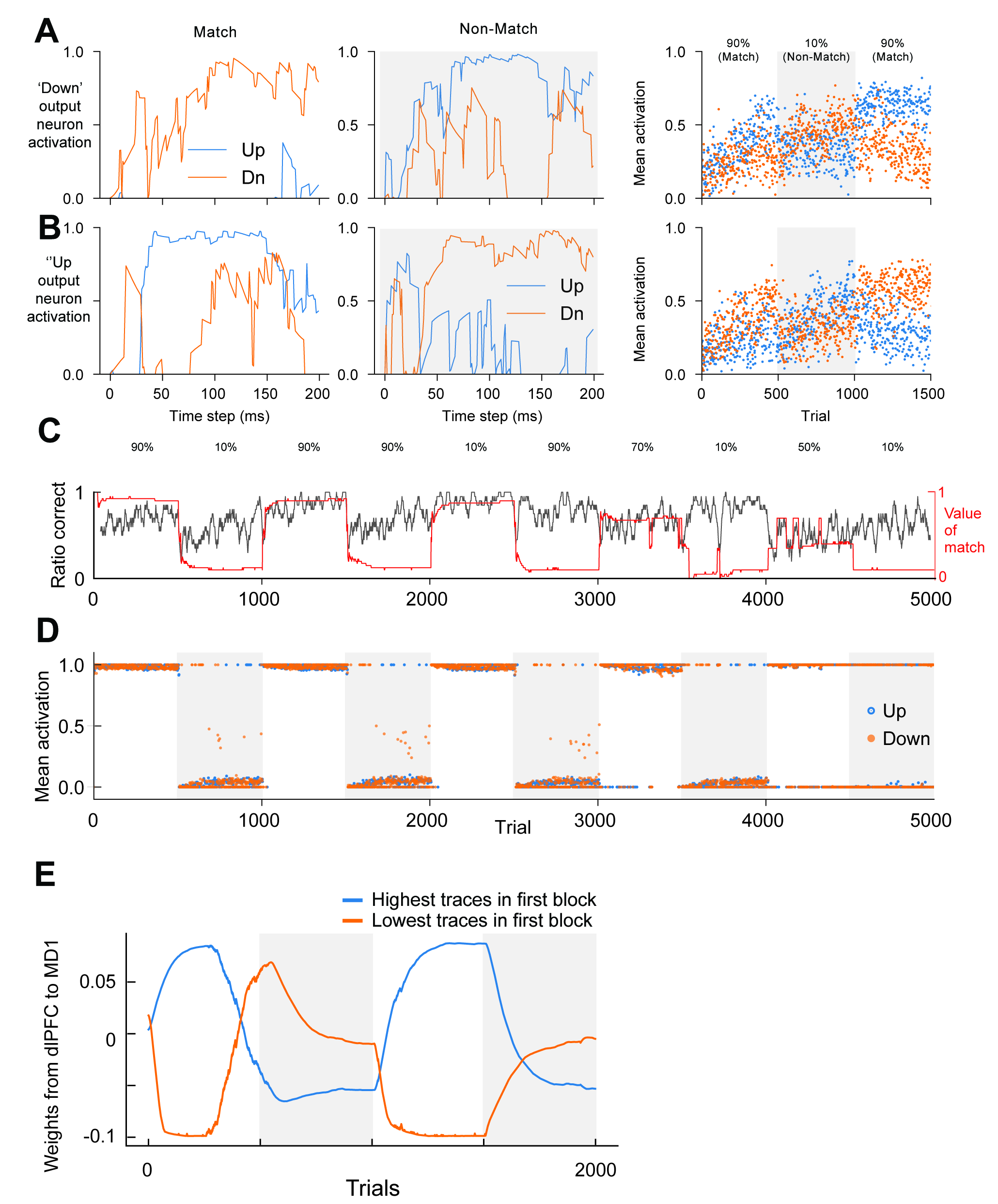

Supplement: S2 Fig — A. Responses of the ‘Down’ output neurons during a trial with input sensory cue as ‘Up’ or ‘Down’, first in a match context (left), then in a non-match context (middle), and trial-averaged activity of the same output neuron over the first three block of the experiment (right). Output neuron activity to the correct responses separates with readout weight learning over the first three blocks of the experiment and correctly reads out target output from dlPFC activity. B. Same as in A but for the ‘Up’ output neuron. C. Behavioral responses of the model across an experiment starting with 90% and 10% blocks, but then including a 70% and a 50% association level blocks. D. Responses of one of the MD neurons with some increased responses in the opposite context when the association level is less predictive. E. Weights from dlPFC neurons to one MD neuron, averaged over the 5 neurons with the highest eligibility trace in block 1 (blue), or the 5 lowest (orange) showing opposing weight dynamics across blocks. (TIF) [file pcbi.1010500.s004.tif]

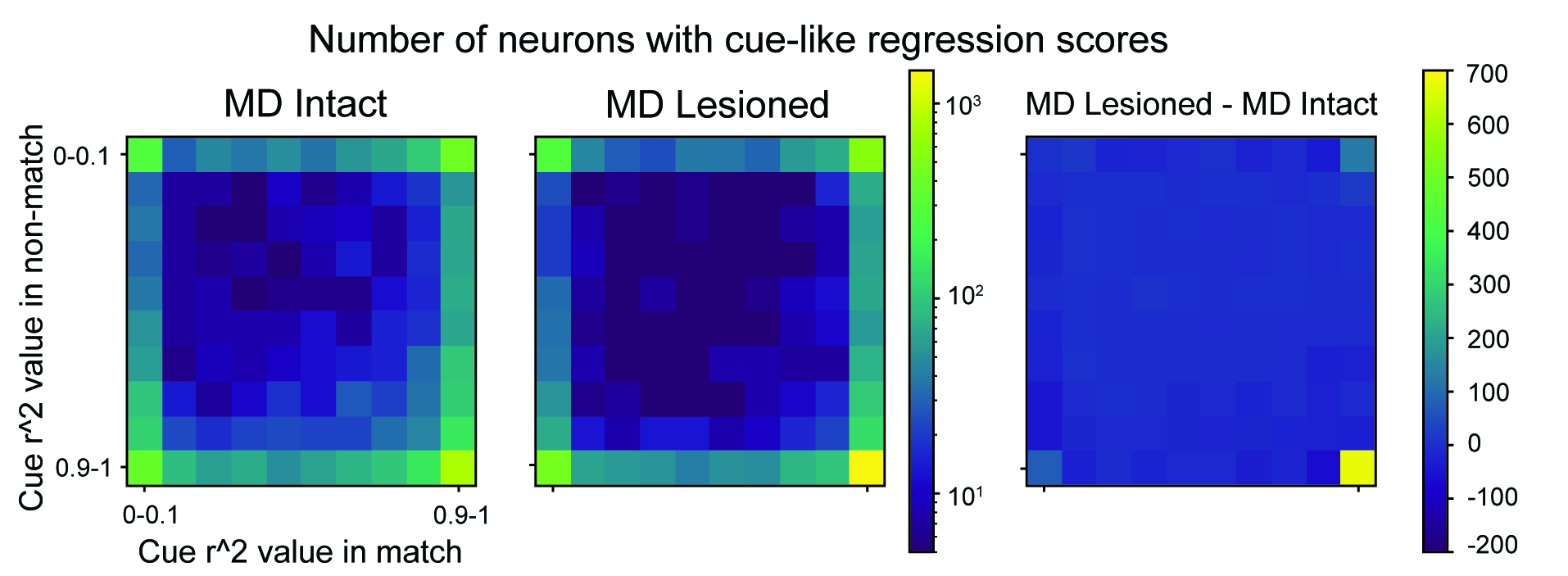

Supplement: S3 Fig — A. Histogram of correlation values (pseudo R-squared) between individual neuronal activity and input cue, with trials drawn from a match context on the x axis, and a non-match context on the y-axis (See Methods, ‘Finding cue-responsive neurons with logistic regression’). The histogram for the MD intact model (left) and MD lesioned model (middle) were subtracted to highlight the differences (right), showing mainly fewer cells that correlated with input cue in both contexts. (TIF) [file pcbi.1010500.s005.tif]

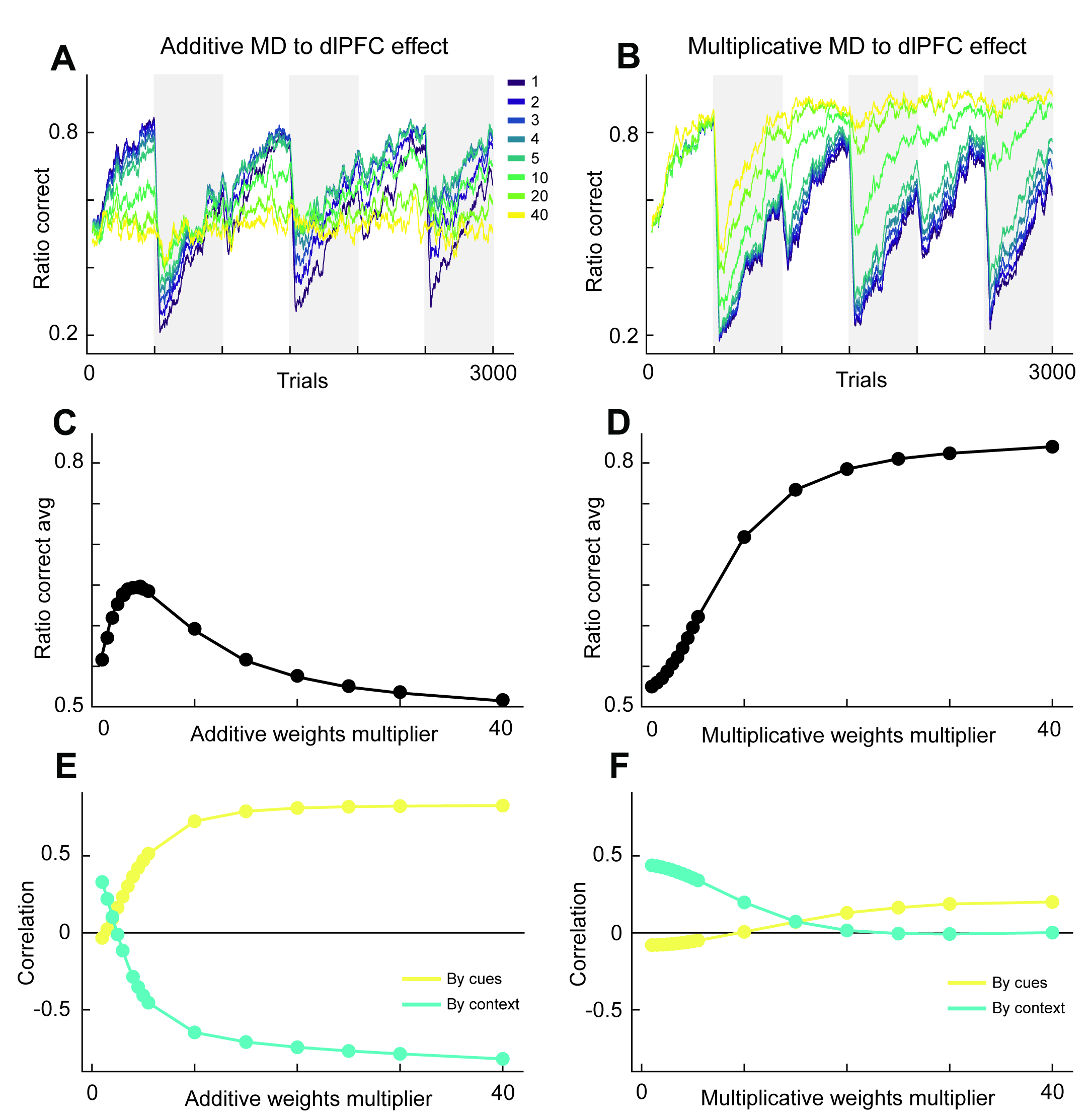

Supplement: S4 Fig — Experiments with alternating blocks of 10% and 90% of match trials rewarded with no vmPFC inputs, but one MD neuron artificially activated for each type of block and Hebbian learning at the corticothalamic projections disabled. We tested the model with multiplicative or additive thalamocortical projections separated and parametrized the strength of either projections by multiplying their respective values by a factor from 1 to 40. A. Model performance for selected strengths of additive projections and B. multiplicative projections. C. Increasing the strength of additive projections initially improves performance and behavioral flexibility at block changepoints but performance rapidly peaks and declines. D. Increasing the strength of multiplicative projections consistently increases performance until reaching 0.9 ratio correct steadily with minimal dips at block changepoints. E. Increasing additive projections strength decreased neural activity correlation in dlPFC for match vs non-match contexts, but also rapidly increased correlation between up and down trials until they become highly correlated, and presumably difficulty to decode. F. Increasing multiplicative project strengths decreases neural activity correlation in dlPFC between contexts with limited increase in correlation between cues. (TIF) [file pcbi.1010500.s006.tif]

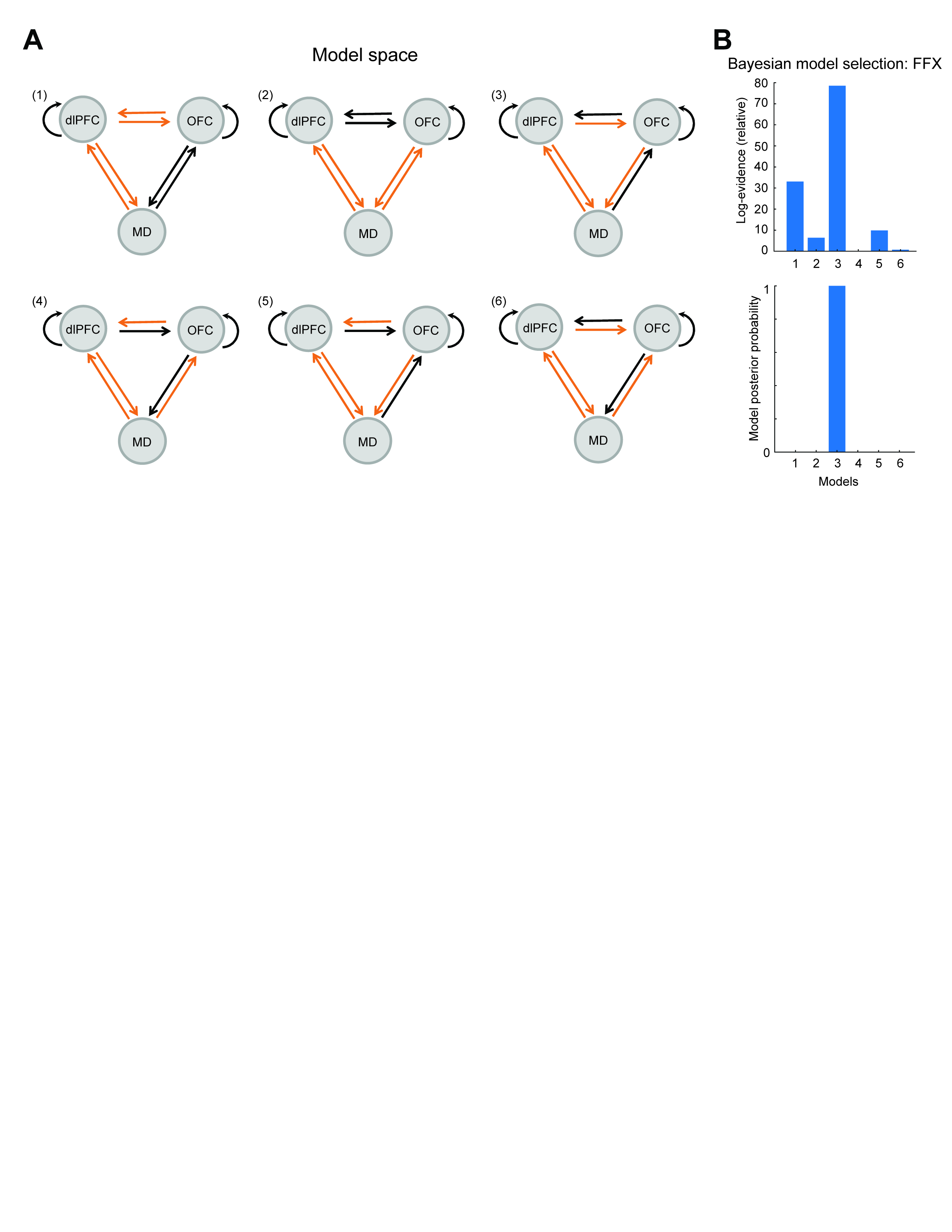

Supplement: S5 Fig — A. Illustration of the model space for Bayesian model selection. We specified six models to determine whether the feedforward, feedback or both connections between OFC were modulated by the strategy switching. We constrained the space to models with assumed dlPFC to MD reciprocal connections as the role of these connections have been demonstrated in animal studies [21]. Bayesian model selection revealed that among the models with the tactile input directed to dlPFC, model 3 was superior to the other 5 models. B. The log-evidence and posterior probability for each of the 6 models. (TIF) [file pcbi.1010500.s007.tif]
