# Supplementary material for: Thalamic regulation of frontal interactions in human cognitive flexibility
Source: PLoS Comput Biol. 2022 Sep 12;18(9):e1010500. doi: 10.1371/journal.pcbi.1010500 (PMC9499289; doi:10.1371/journal.pcbi.1010500)
Supplement: Attachment — Submitted filename: Response to reviewers.docx [file pcbi.1010500.s008.docx]

**We sincerely thank the reviewers and editors for their constructive and helpful feedback. Based on their input, we have done a major revision in which we performed new simulations and analyses and rewrote many sections of the manuscript. We feel that this made our message sharper, and the work more impactful. We hope this revision is satisfactory but remain open to further input.**

**Overview of changes:**

1. **We re-wrote the introduction to increase the scholarly framing of the role of mediodorsal thalamus in cognition, and included more studies that support this general idea.**
2. **We clarified the impression of a lack of focus on dlPFC in our earlier manuscript (Wang and Pleger 2020). That manuscript used a coarser method of spatial smoothing and whole-brain analysis, therefore, the approach was not geared towards distinguishing between different prefrontal areas in the manner that we do now. We explain this point both in the manuscript and the point-by-point response to the reviewers’ comments below.**
3. **We have taken additional care in revising the introduction of the neural model so that the non-experts are able to follow the design principles and their relation to neurobiology.**
4. **We added new simulations to clarify the mechanism by which the MD contributes to initial task learning, and also the effects of MD perturbation after initial task learning. The experiments now show that MD lesioning generates transient deficits that can ultimately be overcome but with significantly prolonged training. This finding solidifies the conclusion that thalamocortical interactions are required for efficient and flexible task performance. This is now reported in an expanded Figure 3.**
5. **We have also made efforts to answer the thrilling question raised by reviewer#3 whether it's possible to map model outputs on human fMRI time series. We intensively discussed this point. In principle, the model outputs a generic average behavior, while participants vary at an individual level when they switch their decision strategies, even in response to the same sequence of trial outcomes. As such, the model latent variables will not correlate with individual brain fMRI recordings, and we leave such analyses to future work with a more suitable dataset.**

**Below, please find our point-by-point response to the reviewers’ comments.**

Reviewer #1: Summary of the research and overall impression

This study aims to translate to humans the past research lines carried out on the rodent model: the computational modeling of the role of the mediodorsal thalamus (MD) on prefrontal cortex interactions during decision making. This is ambitious as the human prefrontal cortex is significantly more complex with debated roles of its sub-territories in decision making, and the MD holds unique primate features not identified in rodents. The authors propose a decision-making task switching across different contingencies inspired by past work on rodent models (changing visual vs. auditory cuing by up vs. down tactile cuing). It allowed them to use the computational model developed in rodents, and expend it in humans. Nicely, the authors report the fMRI activity during their decision-making task, allowing them to identify the orbitofrontal cortex (OFC) and dorsolateral prefrontal cortex (dlPFC) activities to correlate with switching behavior during their task, and demonstrate that MD is engaged in feedback with the dlPFC, and transmit OFC inputs during strategy switching. Overall, this paper is an ambitious and convincing attempt to model the role of MD in prefrontal cortices interactions during the strategy switch in line with contingencies changes during a simple decision-making task in humans. Regarding the thalamus, studies on the role of the MD in humans are mostly lesion studies, which are often non-specific, with a large spectrum of effects on amnesia, dementia, autonomic functions, mood, and sleep/waking cycle. Thalamic nuclei are small and deep structures that are difficult to record with classical non-invasive brain imaging studies, and the few intracranial recording studies focused on memory formation. In this context, proposing a computational model of the role of MD in humans is ambitious, but it brings valuable insights to this largely unexplored domain of research. In particular, this study gives a role to MD that is not being a relay of sensory inputs. To my knowledge, this is the first direct evidence of such a role of thalamus nuclei in humans. Moreover, such models could help to understand the deficits induced by lesions or dysfunction of MD in humans.

**We thank the reviewer for their encouraging comments, and for highlighting this study contributing ‘the first direct evidence for such a role of thalamus nuclei in humans’.**

To me, the outcomes of this study fit the ambition and impact expectations of PLOS Computational Biology. However, if the computation part of the study appears robust and convincing, some clarifications on the selection of the prefrontal regions seem necessary (see major issues below). A more exhaustive report of fMRI results is expected, and the course of the presentation should be rebuilt to show more clearly the unbiased identification of the prefrontal regions of interest.

**We greatly appreciate this comment as it allowed us to go back and clarify some of the critical details surrounding area selection for fMRI analysis. We believe that this has strengthened our manuscript and will undoubtedly increase its impact on the field.**

Major issues

1. The course of the presentation is misleading. While the challenge of the prefrontal complexity in humans is well exposed since the beginning, the literature on sub-territories functions in decision making/task switching is too succinctly introduced and discussed. The dlPFC is given in the introduction as the region of interest for the model with few justifications. Actually, the dlPFC was not reported in the Change>Keep contrast for the same fMRI task in Burkhard Pleger et al., Cerebral Cortex, 2020. Authors should consider using the fMRI data as an unbiased first step of their study to label the entities of their model, and then more extensively discuss this result in line with the existing human literature.

**We very much appreciate the reviewer’s point here, and apologize for not having been clearer in our previous version of the manuscript. The reviewer brings up two distinct points. First, the lack of dlPFC focus in the previous Wang and Pleger 2020 manuscript and second, the appropriate introduction of subregions of the prefrontal cortex in the human brain. We will address these points separately.**

**fMRI data and relation to the previous manuscript. The reviewer is correct in pointing out the lack of explicit focus on dlPFC in the Wang and Pleger 2020 paper.**

**The reason why the dlPFC was not reported in the Change>Keep contrast is largely a technical point rather than a conceptual one. Our analysis in that paper had a different spatial resolution and grouped many prefrontal areas collectively as the premotor cortex. In the current work, we altered our smoothing resolution (specifically, from using a 8mm smoothing kernel to a 6mm one) and paid closer attention to the distinct prefrontal regions. Another important difference is that, guided by the rodent data we constrained our GLM analysis to the prefrontal cortex region, and to the thalamic region.**

**We now clarify our course of thought in approaching ROI in the results section immediately before presenting fMRI results (lines 190-218):**

***“To draw direct parallels between the neural model and human data, we returned to our previously collected dataset [32], and took a different analytical approach from the published work. Mice experiments revealed inhibition of pre-limbic prefrontal region impaired performance and inhibition of MD lead to specific impairment at times when task contingencies changed [21]. As such, we constrained the generalized linear model (GLM) analysis to the prefrontal and thalamic areas, and used a smaller smoothing kernel for improved spatial resolution (see Methods). With this new analysis, we were able to directly evaluate the engagement of different prefrontal and thalamic areas by a small-volume correction [48] (for brain-wide GLM see Tables S1, S2). Within the frontal regions, GLM showed significant activity modulation in dlPFC in trials involving strategy switching (Switching>Staying) ( (t (27) = 7.07, p < 0.001; small-volume FWE-corrected; Fig. 2A), and interestingly, also the orbitofrontal cortex (OFC) (Switching>Staying, , t (27) = 7.31, p < 0.001; small-volume FWE-corrected, Fig. 2A, middle).***

***Within the thalamic region, the MD thalamus was the only thalamic area modulated by switching (Fig. 2A, right, peak MNI coordinates x/y/z =12/−10/8, t (27) = 4.37, p = 0.02, small-volume FWE-corrected). These findings corroborated the involvement of these areas included in the neural model as a direct interpretation of physiological recordings from mice performing a similar task [21], and added the OFC as a region of interest which we introduce to the model in a later section.”***

**We also added this information to the Methods to clarify these differences.**

**Lines 568:**

***“Using the GLM, we investigated neural activity underlying switching the response strategy using the contrast “Switching> Staying”. Based on the previous mice studies and to draw direct parallels to our neural model, we applied a smaller smoothing kernel (specifically, from using a 8mm smoothing kernel to a 6mm one), and we constrained the GLM analysis in the prefrontal areas and thalamus. Therefore, the resulting contrast images for all participants were applied to the group level one-sample t-test and thresholded at p<0.05, small-volume corrected for multiple comparisons at the voxel level (family-wise error rate, FWE) using anatomical regions of interest of the entire prefrontal cortex and thalamus (PFC was defined using the Automatic Anatomical Labeling atlas [86] and thalamus using Anatomy Toolbox [87]).”***

**Scholarly introduction of human PFC subregions. We now add details to the introduction around human PFC subregions and how they would ultimately relate to the different computational modules of the model. Specifically, we state in lines 51-56:**

**“*In humans, frontal cortex consists of several regions that are generally thought to have specialized functions [2–5]. For example, the dorsolateral region is linked to executing cognitive functions [6], while the orbitofrontal cortex (OFC) is associated with adaptive and flexible responding in a changing environment [7]. The ventromedial region is associated with estimating the value and salience of sensory and cognitive variables and thereby, guides value-based decision-making [8].*”**

2. In line with the previous comment, it is unclear if all cortical regions were explored or if the analysis was a-priori constrained to the dlPFC, OFC, and ventromedial prefrontal cortex (vmPFC). For example, it is surprising that activations related to Prior belief appear only in the vmPFC. The table with statistical effect for each cortical ROI is a must-have supplementary element in this study (tables for both Switching>Staying and Staying>Switching contrasts, and Prior belief).

**We thank the reviewer for raising this important point. We constrained our analysis to the prefrontal areas and thalamus. In the revised manuscript, we fully revised the delineation of our a-priori hypotheses to better motivate our ROI approach:**

**Lines 190-201:**

***“To draw direct parallels between the neural model and human data, we returned to our previously collected dataset [32], and took a different analytical approach from the published work. Mice experiments revealed inhibition of pre-limbic prefrontal region impaired performance and inhibition of MD lead to specific impairment at times when task contingencies changed [21]. As such, we constrained the generalized linear model (GLM) analysis to the prefrontal and thalamic areas, and used a smaller smoothing kernel for improved spatial resolution (see Methods). With this new analysis, we were able to directly evaluate the engagement of different prefrontal and thalamic areas by a small-volume correction [48] (for brain-wide GLM see Tables S1, S2). Within the frontal regions, GLM showed significant activity modulation in dlPFC in trials involving strategy switching (Switching>Staying) ( (t (27) = 7.07, p < 0.001; small-volume FWE-corrected; Fig. 2A), and interestingly, also the orbitofrontal cortex (OFC) (Switching>Staying, , t (27) = 7.31, p < 0.001; small-volume FWE-corrected, Fig. 2A, middle).”***

**We additionally provide the results of our brain-wide GLM analyses in the supplement. These other areas, outside our regions of interest, could be contributing to the task and could map onto components of the model which we will continue to explore in future work.**

Supplementary Table 1. Brain regions related to the Strategy Switching (*Switching* > *Staying*) (p < 0.001, uncorrected)

| Regions | Hemisphere | Peak coordinates | | | T-score |
| --- | --- | --- | --- | --- | --- |
|  |  | x | y | z |  |
| Insula | R | 44 | 20 | -4 | 6.89 |
| Insula | L | -30 | 24 | 4 | 9.45 |
| **Dorsolateral prefrontal cortex** | **R** | **46** | **30** | **34** | **7.07** |
| Dorsolateral prefrontal cortex | L | -38 | 30 | 36 | 7.16 |
| Premotor area | R | 34 | 2 | 62 | 7.41 |
| Premotor area | L | -26 | 0 | 62 | 8.03 |
| **Orbitofrontal cortex** | **R** | **28** | **56** | **-6** | **7.31** |
| Orbitofrontal cortex | L | -34 | 52 | 0 | 6.43 |
| Supplementary motor cortex | L&R | 0 | 22 | 46 | 9.40 |
| Inferior parietal lobule | R | 46 | -44 | 56 | 7.95 |
| Inferior parietal lobule | L | -32 | -50 | 42 | 9.27 |
| Precuneus | R | 4 | -62 | 62 | 5.84 |
| Precuneus | L | -10 | -66 | 50 | 7.12 |
| Caudate | L | -10 | 0 | 10 | 6.71 |
| **Mediodorsal thalamus** | **R** | **12** | **-10** | **8** | **4.37** |
| Brain stem | R | 2 | -26 | -24 | 3.42 |

Regions shown in bold indicate the regions of interest in the study (right dlPFC, right OFC and right MD)

Supplementary Table 2. Brain regions correlating with the prior belief in *Switching* and *Staying.* (p < 0.001, uncorrected)

| Regions | Hemisphere | Peak coordinates | | | T-score | |
| --- | --- | --- | --- | --- | --- | --- |
|  |  | x | y | z | |  |
| ***Switching*** | | | | | | |
| **Ventromedial prefrontal cortex** | **L&R** | **4** | **56** | **-8** | | **4.93** |
| Posterior cingulate cortex | L&R | 6 | -48 | 24 | | 5.30 |
| Middle cingulate cortex | L&R | 0 | -10 | 36 | | 4.98 |
| Precentral cortex | R | 40 | -12 | 50 | | 4.01 |
| Middle temporal cortex | L | -60 | -54 | 8 | | 4.35 |
| ***Staying*** | | | | | | |
| **Ventromedial prefrontal cortex** | **L&R** | **-6** | **62** | **2** | | **7.9** |
| Precuneus | L&R | -8 | -52 | 22 | | 6.89 |
| Middle cingulate cortex | L&R | 4 | -12 | 40 | | 6.02 |
| Middle temporal gyrus | L | -40 | -66 | 22 | | 6.77 |
| ParaHippocampus | R | 38 | -28 | -10 | | 5.75 |
| ParaHippocampus | L | -28 | -40 | -12 | | 6.15 |
| Postcentral cortex | R | 58 | -2 | 14 | | 5.72 |
| Postcentral cortex | L | -58 | -6 | 12 | | 5.03 |
| Precentral cortex | R | 22 | -26 | 68 | | 5.35 |

Minor issues

1. The thalamic fMRI data is constrained to the MD, justified by the fact that previous studies in rodents focused on MD, and by the connectivity pattern of the MD. However, the effect in the other limbic thalamic nuclei would have been nice to have, knowing that poor evidence exists yet on the functional differences between those nuclei in humans.

**We thank the reviewer for this point. Our fMRI analysis was constrained to the whole thalamus mask to test whether mediodorsal thalamus is involved based on previous rodent studies. Indeed, we only found the MD activation when we compared switching with staying trials, which indicated MD plays an important role in strategy changes. The MD cluster (peak MNI coordinates x/y/z =12/−10/8) we found mainly connected to the prefrontal cortex based on Behrens TE et al., (2003) Nat Neurosci. 6(7):750-7 (**[**http://www.fmrib.ox.ac.uk/connect/**](http://www.fmrib.ox.ac.uk/connect/)**). No other thalamic nuclei that have interconnections with the limbic system were found in Switching trials from the results of fMRI analysis. It is plausible that the other limbic thalamic nuclei were involved during the task, but they were not cognitive flexibility-related and were canceled out when we compare Switching with Staying trials. Therefore, the limbic thalamic nuclei are not the region of interest in the current study.**

2. The statistical difference between correct predictions in blocks with low predictability compared in comparison to blocks with unpredictability is not reported. The results from the ONE-way ANOVA should be shown in figure 1D. Actually, outcomes from a two-way ANOVA with interaction would be preferable with the type (Human/model) and association level (90-10/70-30/50%) as factors.

**We appreciate the reviewer raising this point about statistical testing. To directly address the reviewer’s comment, we now run a two-way ANOVA (unbalanced design; anovan.m in MATLAB) followed by a multiple comparison test (multcompare.m in MATLAB). We report the statistics of the human/model group comparisons across all association levels and high/low comparisons for human and model in the *Statistics* section of supplementary methods. Specifically, the results are in the following tables:**

| Group A | Group B | P-value |
| --- | --- | --- |
| human/(90-10) | human/(70-30) | < 0.0001 |
| human/(90-10) | human/50 | < 0.0001 |
| human/(70-30) | human/50 | < 0.0001 |

| Group A | Group B | P-value |
| --- | --- | --- |
| model/(90-10) | model/(70-30) | < 0.0001 |
| model/(90-10) | model/50 | < 0.0001 |
| model/(70-30) | model/50 | 0.039 |

| Group A | Group B | P-value |
| --- | --- | --- |
| human/(90-10) | model/(90-10) | 0.990 |
| human/(70-30) | model/(70-30) | 0.990 |
| human/50 | model/50 | 0.101 |

**The text, figure subplot, and legend are now updated with the statistical method and results. Figure 1D (included below) has been updated to depict the statistical results from the tables above.**

3. The authors did a significant and appreciable writing effort. However, they should consider doing an additional rereading effort of their manuscript including spelling, spacing, figure labeling (e.g. figure 1D and 1E and inverted between figure and text), and figures (e.g. missing error bar in figure 1D).

**We apologize for these errors. We have now corrected the mentioned ones but also spent time going through the manuscript more carefully in general. We thank the reviewer for the constructive critical comments.**

Reviewer #2: In this paper, Hummos and colleagues refine a previously developed neural model of an executive frontal-thalamic (MD) circuit based on mice work, and train it on a human decision-making task based on either a matching or non-matching rule on tactile percepts associated with variable contingencies. Both the nature of the rule and the contingencies can be parametrically varied to stimulate flexible responding. As a major take-home, the data suggest that the MD could act as a transthalamic hub to support indirect cortico-cortical communication in humans which may be required when flexible responding is warranted, as also supported by neuroimaging data. Altogether the paper is well written and of high interest for the field and even the broader audience as it effectively stimulates thoughts and proposes new insights to reveal the computational principles at play within thalamocortical circuits, with a possible anatomical relevance. I believe there are a few points that could developed or clarified to increase the value of the paper further.

**We thank the reviewer for their thoughtful comments on our work. We appreciate their support but also recognize the need to do better. We hope that the reviewer finds revisions below and in the manuscript satisfactory.**

1/ The neural model

Some additional descriptions or statements could be provided to better explain some crucial aspects of the model, especially for non-specialists. When considering the main features of the model, it is crucial to distinguish between what is driven by well-established facts from what is actually a working hypothesis.

**The reviewer raises an excellent point, which we very much appreciate. To directly address it, we extensively edited the second section in Results where the model is introduced to the reader, including the motivation for choosing the relevant areas, and the specific anatomical and physiological properties that were considered in modeling them. We include the paragraphs here for reference:**

Lines 143 -177:

***“The neural model: Previous data analysis of fMRI signals in this paradigm revealed multiple interactions between frontal cortical areas and the MD thalamus [32]. To derive insight into putative computational mechanisms, we extended a neural model encompassing the MD and an executive PFC region (Fig. 1B) which had been used to implement a context switching task in mice [21]. Our neural model includes a reservoir of recurrently connected neurons to model the executive prefrontal cortex (dlPFC in primates), consistent with prior modeling efforts in this domain [38,39]. Sensory input (up and down tactile cues) are projected onto the dlPFC (presumably through sensory cortical hierarchy), consistent with recordings of sensory responsive neurons in the mouse prefrontal cortex [21], and analysis of human fMRI detailed below. Responses (up and down) are read out from two output units with the output weights learned through a biologically-plausible learning rule (node perturbation [40], see Methods).***

***Model MD neurons are devoid of local excitatory connections consistent with electrophysiological findings [41] and include a winner-take-all mechanism representing mutual inhibition through the thalamic reticular nucleus [41,42]. Previous work in mouse indicated that the MD thalamus integrates its prefrontal inputs to generate a compressed representation of the task’s context [20,21]. We explored whether such representation could emerge in the model based on a Hebbian learning rule at the dlPFC to MD corticothalamic connections, inspired by recent work [43] suggesting that Hebbian learning mediated unsupervised clustering. Using higher number of MD neurons showed similar results so for simplicity, we kept the number at 2 neurons for the current study.***

***Previous experimental evidence showed that MD thalamic projections engaged direct inhibition of prefrontal neural activity and indirect amplification of local functional connectivity through disinhibitory mechanisms [20,44,45]. We model these experimental observations by an additive (suppressive) and multiplicative (amplifying) thalamic output to the prefrontal reservoir recurrent neural network (RNN). Model behavior did not require learning at these projections and they were chosen randomly and held fixed for stability.***

***A key difference between the previous animal work upon which our neural model was designed and the current task is that inferring a block switch in the current task requires a record of recent rewards. So, we provided the model with inputs representing the value of the two behavioral strategies in recent trials (i.e., expected rewards from match vs. non-match strategies). These value inputs improved model behavioral flexibility and provided a signal to disambiguate the two contexts based on recent reward patterns (Fig. S1A-D). Consistent with this modeling assumption, fMRI data of humans performing the same task showed that vmPFC activity correlated with prior belief about outcome value, indicating the availability of such signals in the prefrontal region (Fig. S1E, F, also see [46,47]).”***

For example, the rationale that the MD exhibits “winner take-it-all” computational properties should be better explained. Is this based on empirical facts or a computational hypothesis? The reference provided l.148 based on anatomical work from Pinault does not seem to support this statement.

**We thank the reviewer for this comment. Winner-take-all design principle is equivalent to mutual inhibition, which is implemented through the TRN. The Pinault review illustrates this point (Fig. 3), but the original work is by Barry Connors’ lab, which we now cite.**

Lee S-C, Cruikshank SJ, Connors BW. Electrical and chemical synapses between relay neurons in developing thalamus. J Physiol. 2010;588: 2403–2415. doi:10.1113/jphysiol.2010.187096

**The changes to the manuscript are as follows**

Lines 154-162:

***“Model MD neurons are devoid of local excitatory connections consistent with electrophysiological findings [41] and include a winner-take-all mechanism representing mutual inhibition through the thalamic reticular nucleus [41,42].”***

There are only 2 neurons considered for the MD in the model so is this a limit of the current model or one of its feature to implement the “winner take-it-all” rule?

**We now elaborate on this important issue further. The use of two neurons was all that was required for successful running of the current tasks, so we limited the main presentation to that version of the model. We have run simulations with a higher number of MD neurons but they effectively cluster into two groups that implement the function of the two neurons, at least with the current tasks used. So for simplicity, we limited the model MD neurons to 2.**

**We now added a sentence as we introduce the model to clarify:**

**Lines 160-162:**

***“Using higher number of MD neurons showed similar results so for simplicity, we kept the number at 2 neurons for the current study.”***

In a similar way can we really make meaningful functional comparison between the 500 neurons dlPFC reservoir and the 2 neurons MD? See line 353-354 for one instance of the latter.

**As we indicate above, the 2 neurons for MD emerged from the fact that the task can be solved by two dominant strategies either to match or non-match the sensory input. We reformulate our sentences now to focus on the underlying conclusion that MD will have a more compressed representation of the current behavioral state. (If not due to the compressed representation we propose, then just by a shear lower number of neurons). And interfacing with this low dimensional representation is more efficient. We think that holds regardless of the specifics of our model. We now restate the sentences to focus on those ideas.**

**Lines 362-364:**

***“While this result depends to an extent on our proposal that MD has a more compressed representation of the task context, these trends would also hold due to MD having fewer number of neurons.”***

I was also a bit confused on the statement regarding the RNN which “did not have a mechanism to encode recent rewards” (the paragraph l.161-165). It is then said that the model is provided with inputs representing the value of each rule “computed over recent trials”. But if it is computed over recent trials what is then the difference from having a memory of recent rewards? Please clarify the difference.

**Thanks for pointing out this potentially confusing statement. “Computed over recent trials” was used to indicate that we ourselves computed them. We now simply state what is relevant here, that we supply value estimates as inputs to the model. We hope it is clearer and more succinct.. We now restated the paragraph to clarify as follows:**

**Lines 169-174:**

***“A key difference between the previous animal work upon which our neural model was designed and the current task is that inferring a block switch in the current task requires a record of recent rewards. So, we provided the model with inputs representing the value of the two behavioral strategies in recent trials (i.e., expected rewards from match vs. non-match strategies). These value inputs improved model behavioral flexibility and provided a signal to disambiguate the two contexts based on recent reward patterns (Fig. S1A-D).”***

The data illustrated in figure 2C need to be more comprehensively described and analyzed. What do top and bottom panels represent exactly, what is the difference between the two and how does this ultimately support the conclusion that dlPFC, not OFC or MD acts as input region? There is only one short mention in the main text l.207-208 which is insufficient to understand this and it is crucial for the next parts of the paper.

**We appreciate the reviewer’s comment. We re-organized Fig. 2 and added more information to introduce the results of DCM causal pattern selection in the main text and the legend of Fg.2.**

**Lines 224-232:**

***“BMS evaluates the evidence that compromises the accuracy and complexity of a causal model and uses the group log-evidence (which is equivalent to the log group Bayes factor) to quantify the relative goodness of fit. The sum of the log-evidence over subjects can be used to compute how likely it is that a specific model generated the data at the group level (i.e., the expected model posterior probability). Consistent with the neural model construction and a direct extrapolation from previous animal work [21], we found that the model posterior probability revealed very strong evidence in favor of the causal model in which sensory inputs first enter the dlPFC-MD-OFC circuit through the dlPFC (posterior probability = 1.00, Fig. 2C).”***

**The legend of Fig.2:**

**“*The left panel shows the average and standard error of log-evidence for all models in the corresponding inputs family (we have 6 models for each input family, see Fig. S5). The sum of the log-evidence over subjects was used to compute the expected model posterior probabilities of each input family which are shown in right panel*”**

Finally, I think it would have been great to discuss a little further the extent to which the proposed model also has anatomical relevance. I think it is relatively well described that the OFC is the recipient from multiple sensory streams (e.g. classic neuroanatomy work from Cavada et al., Cerebral Cortex 2000), consistent with a major integrative role for this region. So I expect that some neuroanatomists would have expected that inputs may be conveyed from the OFC to the dlPFC rather than the opposite. This at least should deserve a more thorough discussion. Perhaps views from Barbas & colleagues could also be considered here to strengthen this aspect (e.g. García-Cabezas & Barbas, 2017). In direct line to this, the final paragraph l.358-366 is very short and do not bring much data for the reader to help consider the relevance of the proposed model (inputs reach dlPFC first, not OFC or MD). This part would benefit from being a little more scholar with clearer explanations of the data considered and the types of analyses that have been done.

**We thank the reviewer for raising this point and the citations provided. As detailed in the response to comment above, we now carefully introduce the methodology and elaborate on these findings. Additionally, we now include a discussion of these relevant views directly in the results section due to its importance in interpreting our findings.**

**Lines 234-241:**

***“In addition, this finding also relates to controversies about the direction of communication between the dlPFC and OFC where some evidence points to the OFC as the recipient of multi-modal sensory input [49] and thereby, likely forwarding such information to dlPFC, while other evidence points to OFC being hierarchically higher and contextualizing representations in dlPFC [50]. Our findings are in support of the later view, at least in this task. Our analysis did not include lower-level sensory areas but focused on the subgraph of the three areas implicated in cognitive flexibility in this task.*”**

2/ Perturbation experiment

The inclusion of a perturbation approach is nice and could be very informative to test some of the predictions of the model. In its present form however, it is difficult to have a clear functional understanding of MD deletion in the RNN. The data presented on figures 3F-I need to more thoroughly explained and analyzed. At present the main text and the caption do convey ambiguous statements. The main text says that the initial effect of MD lesion on flexible switching became less prominent after further training (l.233-234) but the caption of figure 3 (l.253-254) state that initial blocks showed no lesion effect unlike subsequent blocks which suggests the opposite. So I don’t really understand the nature of the deficit here. A transient deficit or acquisition impairment is a common feature in the rodent literature documenting the effect of MD lesion and has been relatively widely been discussed (there are multiple reviews out that document this specific issue) and it would be nice to compare that with the present dataset. I think the effect of MD lesions really need a much clearer description on what exactly is being impaired, what is not, and whether any of the effects are permanent or temporary.

**The reviewer’s comment inspired us to consider more in depth analyses and perturbation experiments. But first, to clarify the conflicted statements the reviewer refers to: the first statement referred to the very first block where both models (MD intact vs. MD lesioned) saw only one sensorimotor mapping and learned it exactly the same. Under these conditions, deficits in the MD-lesioned model started after a context switch was introduced. The later statement referred to the fact that even without an MD, the cortical model eventually learns the two contexts and how to switch between them, but requires considerably longer training time. The deficits we see are early on in the learning process, and also limited to the trials after a block switch (Panel G below). We hope this clarification helps with the confusion generated in the previous version of this manuscript.**

**In order to directly address the reviewer’s larger point, we expanded our study and introduced a new simulation that lesions the MD following task acquisition (multiple block learning). This lesion also generates a transient disruption in switching (Panel H below), which is consistent with the animal literature that the reviewer is pointing out. We thank the reviewer for encouraging us to clarify this point as it now connects the dataset from our simulation to the broader thalamocortical neurobiology literature in a direct way.**

**Of note, we now also quantify the statistical significance in each block using a t-test and display it on the figure for clarity.**

Given the scope of the paper, it would be expected to compare that with actual lesion data, there is a myriad of those available both in rodent and NHPs. This may help to strengthen the relevance of the model further and/or to identify some limits.

**The reviewer is correct and given the two simulations we have pointed out in the previous comment, we hope that they fully address the current point. We have referenced the following lesion studies in rodents and non-human primates in the new manuscript.**

*Wolff M, Faugère A, Desfosses É, Coutureau É, Marchand AR. Mediodorsal but not anterior thalamic nuclei lesions impair acquisition of a conditional discrimination task. Neurobiol Learn Mem. 2015;125: 80–84. doi:10.1016/j.nlm.2015.07.018*

*Alcaraz F, Naneix F, Desfosses E, Marchand AR, Wolff M, Coutureau E. Dissociable effects of anterior and mediodorsal thalamic lesions on spatial goal-directed behavior. Brain Struct Funct. 2016;221: 79–89. doi:10.1007/s00429-014-0893-7*

*Alcaraz F, Fresno V, Marchand AR, Kremer EJ, Coutureau E, Wolff M. Thalamocortical and corticothalamic pathways differentially contribute to goal-directed behaviors in the rat. Elife. 2018;7: e32517. doi:10.7554/eLife.32517*

*Fresno V, Parkes SL, Faugère A, Coutureau E, Wolff M. A thalamocortical circuit for updating action-outcome associations. Behrens TE, Schoenbaum G, Schoenbaum G, Corbit L, editors. eLife. 2019;8: e46187. doi:10.7554/eLife.46187*

*Ouhaz Z, Ba-M’hamed S, Mitchell AS, Elidrissi A, Bennis M. Behavioral and cognitive changes after early postnatal lesions of the rat mediodorsal thalamus. Behavioural Brain Research. 2015;292: 219–232. doi:10.1016/j.bbr.2015.06.017*

*Alcaraz F, Naneix F, Desfosses E, Marchand AR, Wolff M, Coutureau E. Dissociable effects of anterior and mediodorsal thalamic lesions on spatial goal-directed behavior. Brain Struct Funct. 2016;221: 79–89. doi:10.1007/s00429-014-0893-7*

*Wolff M, Vann SD. The Cognitive Thalamus as a Gateway to Mental Representations. J Neurosci. 2019;39: 3–14. doi:10.1523/JNEUROSCI.0479-18.2018*

*Chakraborty S, Kolling N, Walton ME, Mitchell AS. Critical role for the mediodorsal thalamus in permitting rapid reward-guided updating in stochastic reward environments. In: eLife [Internet]. eLife Sciences Publications Limited; 2 May 2016 [cited 2 Jun 2022]. doi:10.7554/eLife.13588*

**Lines 292-295:**

***“These impairment patterns are consistent with animal experiments where MD inhibition delayed the acquisition of tasks requiring flexible responses [23,24,54–56], but eventually MD inhibited animals achieved normal performance after extensive training [23,24,57].”***

3/ Context vs rule

I was confused by some of the terminology used in the paper and in particular the use of the term ‘context’ which I did not expect to be necessary here. Both ‘temporal context’ and ‘behavioral context’ are used in the ms to describe the fact that the rule can change (matching vs non-matching). The first occurrence of this is line 218 and I really don’t understand the rationale for labelling this as ‘temporal context’ when what is really meant here is that MD neurons may encode the current rule within a block. Introducing ‘context’ here seems unnecessary and actually incorrect since there is no element to signal a context. Adding ‘temporal’ (and then ‘behavioral’ and multiple further instance) only makes the issue even worse and obscure much of the interpretation. I would really suggest to remove these terms throughout the whole ms or to better explain the rationale for using them (and especially what it brings to use those). To identify the current rule, participant must track any difference between expected and actual outcome but I really don’t see how the context could play a role in this.

**The reviewer makes an important observation that we ended up spending significant time considering and debating and it helped us clarify our terminology. We apologize for not properly grounding these terms. We now define our intended meaning by ‘context’ and ‘temporal’ and we discuss our rationale for using these terms here.**

**Briefly, rules in this task map to “matching” vs “non matching” the current sensory input. Participants see a combination of these two rules in each block depending on the association level (90%, 70%..etc). What changes over time is not the rules themselves but their proportions in each block, which we interpret as context.**

**The reviewer is also correct to point out that there are no contextual signals here, but participants rather had to infer the context probabilistically.**

**Rule: input-output transformation or mapping between a sensory cue and a cognitive outcome**

**Context: a variable defining the proportion of rules that varies across blocks (time).**

**We now explicitly define these terms in lines 108-113:**

***“Participants responded by either matching the input cue (match rule) or responding with the opposite direction (non-match rule). The task consisted of unannounced pseudorandom blocks with different association levels between rule and rewards, which we refer to as distinct contexts requiring different sensorimotor mappings akin to the rodent work [21]. Contexts had cue-reward associations that were strongly predictive (90% of match responses rewarded, or 10%), moderately predictive (70% and 30%) and non-predictive (50%).”***

**And lines 249-253:**

***“In comparison, neural model MD neurons encoded the dominant strategy within a block, which we interpret as a temporal context, as it is not signaled by a contextual cue but rather can be inferred from its structure in time ”***

I have a few more minor comments.

1. I think that the data considered in the introduction and the whole rationale to present the focus on the MD is a bit thin. There is a lot of literature out there and only 2 MD papers are considered. It should be easy to build a stronger case considering the vast literature available. Similarly, I found the initial question l.71 to introduce the present work a bit narrow as it only asks whether the MD could mimic Pulvinar functions on frontal systems. The Pulvinar surely is a high-order thalamic nucleus like the MD but unlike the MD it is modality specific so I think the MD is a candidate to have actually possibly broader (even more integrative) functions, at least across modalities. I think it would impact more significantly on the general audience to better introduce the present work and how it is positioned to influence what is now a quite dynamic field, with important functional questions.

**We agree that our introduction could have been broader and we appreciate the reviewer’s comments. We now focus our introduction on the MD and expand more on its extensive connectivity both to the prefrontal cortex and to many regions in the brain, highlighting a central role in cognition. The reviewer is correct in pointing out that a part of the Pulvinar is involved largely in visual processing (inferolateral part). To directly address the reviewer’s comment, we cite more studies highlighting the functional and anatomical relevance of the MD to prefrontal cortices and cognition. The introduction paragraph has been restated to read:**

**Lines 66-81:**

***“Specifically, such studies of associative thalamic regions have indicated a role for this subcortical collection of excitatory neurons, devoid of recurrent connections, in sustaining, switching, and synchronizing connected cortical areas [15,20–24]. For example, in mice, multiple studies have shown that the mediodorsal thalamus (MD) projects to areas of prefrontal cortex with signals able to sustain or switch task-relevant activity patterns, enabling the maintenance of working memory on one end and task switching on another [20,21]. The anatomical divergent and convergent cortical projections originating from the thalamus makes the thalamus an ideal location not only for information integration but also deployment of simultaneous instructions [25,26]. Specifically, studies have shown diffusive projections from neurons in MD thalamus across several areas of prefrontal cortex in rodents [27–29,29,30]. Beyond the MD’s dense connections with frontal cortical regions, the structure also receives projections from regions within the temporal cortex, the midbrain, and the basal ganglia [16,27,31], positioning it well to have a broad integrative function. Given the studies above, two questions are pertinent: first, is the MD thalamus a functional mediator and a communication bridge for frontal cortical areas? Second, what are the precise computational functions of such engagement?”***

2. The mentions on the non-relay functions of the MD may appear a bit trivial in 2022 (e.g. l.78, l.202, l.205, and especially l.209). Surely we knew it before the present work was conducted that the MD is not relaying tactile sensory information to the prefrontal cortex. Consider the sentence l. 208-209; alternatively, what would be the evidence demonstrating instead that a thalamic area actually engages in a task in humans to relay sensory information? I don’t think this bring much to main topic of the work and it is possibly distracting.

**We appreciate this point, and have softened the sentence under question as follows:**

**Lines 232-234:**

***“While the non-relay functions of thalamic nuclei in the human brain are suspected, evidence remains indirect and, to our knowledge, this is one of the few direct demonstrations that a human thalamic area engages in a task not to relay sensory inputs.*”**

To get back to the previous point, I even wondered upon reading to this why the authors did not look at the Pom instead (the high-order thalamic nucleus for somatosensory information) as an actual parallel to the Pulvinar (especially l.205)?

**The reviewer is correct that the inferolateral pulvinar may be similar to POm. Importantly though, the PoM did not show a significant engagement in this task, in contrast to the MD which became the focus of the study. We now introduce the MD in a broader perspective and avoid the analogy to the Pulvinar completely.**

3. Considering the model was initially developed to account for the performance of mice in a cross-modal attention task, I think it would be great to compare more clearly the mouse task with the human one as they have a number of clear differences which may impact at the functional level (e.g. cross-modal versus purely tactile task, cue signaling the current rule versus need to monitor the difference between expected and actual outcomes to identify the current rule, mice learn everything by trials-and-errors while participants are warned they will be rule changes – even though the changes are not directly signaled etc…).

**This is a great point. We have included a paragraph in the discussion section to highlight the task differences (lines 422-430):**

***“In addition, in comparison to the mouse cross-modal attention task, upon which the model was based, the human task was a purely tactile task, whereas the mouse task is cross-modal, requiring the animals to attend to auditory and visual cues. In the mouse task, the current rule is directly signaled per trial and requires no inference, while in the human task, rules were inferred from monitoring of expected versus actual outcome. Further, humans were instructed that rule changes may occur (although unannounced), whereas animals had to learn the task structure from trial-by-trial errors. Despite these differences, we found that the extended rodent model reliably captured human behavior, emphasizing the robustness of our model. “***

4. In the discussion, the rationale for the statement l.437 that “thalamocortical connections are either fixed or updated at a much lower rate compared to corticothalamic projections” is not provided or explained. Please expand here. Is the fact that CT projections typically outnumber TC projections relevant here?

**We now expand on this point and discuss the need for fixed thalamocortical projections and interpret how that might relate to brain mechanisms (Lines 477-484):**

***“Our neural model used fixed thalamocortical connections and successfully captured behavior and flexible contextual switching, indicating a potential architectural design where the thalamocortical connections are either fixed or are updated at a much lower rate compared to corticothalamic connections. We initially reasoned that Hebbian learning at the thalamocortical synapses would induce formation of modules in the RNN, but in practice it created an echo chamber where the corticothalamic synapses learned an association and the thalamocortical synapses reinforced it, preventing the model from switching to other states, indicating that the brain might have special solutions for learning reciprocal projections between two areas. “***

5. Some typos: l.139 perhaps a ‘to’ is missing, l.157 ‘disinhibitory’

**Fixed. Thanks**

Reviewer #3: The manuscript by Hummos and colleagues provides a modelling study investigating thalamocortical circuit mechanisms of context-dependent learning. This is combined with both behavioural and fMRI data from a human learning experiment, to which the model was applied. DCM on the fMRI data is used in attempt to arbitrate between different model architectures. In the task, participants are presented with a tactile stimulus (up or down) to which they have to respond with a match- or non-match response. The cue-response mappings (match- vs non-match) are probabilistic and change between blocks of trials (0.9, 0.7, 0.5 probability of the match- vs non-match response being correct).

The neural network model had four components: dlPFC (modelled as RNN with reservoir dynamics), mediodorsal thalamus (MD), vmPFC (providing value-related input), and OFC (providing estimates of the probability that the underlying latent state has changed). Importantly, thalamocortical connections (MD--> dlPFC) were fixed, while corticothalamic (dlPFC-->MD) weights undergoing Hebbian learning. The model behaviour closely tracks human choice data and model vmPFC output closely follows the underlying action probabilities. fMRI results show increased activity on strategy swith vs stay trials in dlPFC, MD, and OFC. DCM applied to these fMRI data supports a model in which sensory inputs first enter dlPFC, rather than being relayed via MD.

There are several results in this paper that I found very interesting. The authors investigated the coding properties of model dlPFC and MD neurons. In line with previous experimental findings, the architecture gave rise to mixed selectivity in dlPFC neurons. In contrast, MD neural activity was tightly related to the current context, or block (match vs non-match). Notably, the latter property of MD neurons relied on the long time scale of the Hebbian eligibility trace. When this time constant was reduced, MD neurons were biased to tracking the sensory signal (up vs down cue) that varies from trial to trial.

**We are thankful for the reviewer emphasizing how these experiments with the Hebbian time constant are integral to the story, and we moved these results from the supplementary material to main figure 3.**

Learning data of a model with MD lesions (output of MD to dlPFC removed) showed a "reversal learning deficit" - the lesioned model took longer to learn the new contingencies following a covert block switch, but it eventually reached control levels within a few hundred trials. The MD lesion also reduced cue selectivity in dlPFC neurons. Furthermore, in the intact model, connections between dlPFC reservoir to output neurons for the appropriate context increased for the correct context, but remained silent out of context. Finally, DCM results provided evidene in favour of transthalamic routing of OFC signals (OFC signalling to dlPFC via MD, rather than direct cortio-cortical signaling).

As stated above, I think this manuscript provides important new insights regarding the thalamocortical circuit mechanisms underlying context-dependent learning/probabilistic inference. I should point out that I am not an expert in neural network modelling (far from it), so I cannot provide a well-founded judgement on these methods. My comments below also mainly pertain to clarifying some questions that came up while reading the manuscript.

1) I think the cartoon of the model in figure 1B should be elaborated. The neural network model takes centre stage, so this needs to be clear. Before I arrived at the methods, it had not been really clear to me which components the model included - and which brain areas they were thought to represent. For example, I initially thought that the authors used vmFPC and OFC interchangeably, until I realized that these were separate components. Perhaps it would even be an option to have a separate figure solely dedicated to the model (and the variations of it that were used) to make the approach more straightforward to follow. Questions on the architecture that still remain open to me:

- How exactly does the MD component of the model look? I think that it contains two excitatory neurons that project to a population of inhibitory cells (hypothesized in the reticular nucleus) that provide feedback inhibition - is this correct?

**We thank the reviewer for making this critical clarification. We now redesigned figure 1 to show the MD model in more detail. Correct, two neurons were sufficient, and the winner-take-all mechanism is modeled as mutual inhibition. All depicted now in the subpanel attached below.**

**Figure 1B:**

- The OFC component is (at least what I understand) not at all present in the schematic (1B) - was it added only at a later stage?

**Yes, the reviewer is correct in that the OFC component was added to expand the capability of the model in later experiments. We now make this transition more explicit and we added a sentence marking where the OFC component was added and why:**

**Lines 339-341:**

***“Analysis of human prefrontal fMRI revealed engagement of OFC in the network switching motif (dlPFC, OFC and MD), therefore we extended our model with a module representing OFC computations, to gain further computational insight into the role of the MD in possible OFC-dlPFC interactions. “***

**We also indicate earlier on that OFC module would be added later in the study:**

**Lines 215-218:**

***“These findings corroborated the involvement of these areas included in the neural model as a direct interpretation of physiological recordings from mice performing a similar task [21], and added the OFC as a region of interest which we introduce to the model in a later section.”***

- Is there anything about the RNN architecture that would make it particular to the dlPFC? In other words, could this component map to any cortical area (with strong recurrent connectivity)? Did the mapping of the component to dlPFC result from the fMRI findings?

**The reviewer raises a good point about the generality of the computational framework we examine. We agree that the RNN model is quite general and can be applied to most cortical areas. The reviewer is correct, the rodent data constrained our interest to the prefrontal region, and fMRI analysis highlighted the dlPFC. There is also a precedence for using reservoir RNNs to model the dlPFC (Enel et al., 2016).**

Enel P, Procyk E, Quilodran R, Dominey PF. Reservoir Computing Properties of Neural Dynamics in Prefrontal Cortex. PLoS Comput Biol. 2016;12. doi:[10.1371/journal.pcbi.1004967](https://doi.org/10.1371/journal.pcbi.1004967)

2) I was intrigued when looking at the model MD neuron firing patterns (Figure 3D) - this looked to me like a code that very sharply represents the latent task state - thus holding a representation very similar to those found in OFC (Wilson et al., Schuck et al.). I have difficulty understanding how the representation found here would differ from an OFC representation of latent states. The MD representation seems to be very sharp (apparently emerging immediately after a block switch) and binary (where I would assume the latter part results from the winner-take-all dynamics in MD?). Could you please elaborate on how this emerges (in particular so abruptly) - is it a representation that is inherited from the OFC component, which I assume will be similarly sharp (threshold crossing of the switch probability)?

**We thank the reviewer for raising these questions. The model MD generates a latent variable representation based on dlPFC inputs, and it is not inherited from the OFC. This is based on the biologically-plausible learning rules on convergent dlPFC-to-MD connections. In fact, when the time constant of this learning rule is shortened, the latent representation vanishes (Fig. 3F, below). We now moved this result to the main manuscript to address the point the reviewer brings up. We suspect that in the brain, these latent variables are simultaneously computed in multiple distributed regions, and we hope to explore this topic in future modeling projects.**

3) The fMRI results (with the exception of the DCM) are somewhat touched only quite briefly. In particular, I had been wondering what the rationale was for using a simple switch>stay contrast as the main fMRI contrast (which yielded dlPFC, OFC and MD; figure 2). I think this could be elaborated a bit in order to relate the regions more to their underlying functions as specified in the model. Wouldn't it be interesting to use e.g. the output of the OFC component as regressor in the GLM to test whether this region truly tracks the underlying latent state? Similarly with the model MD output (possibly also dlPFC). I appreciate that the dominant focus of this manuscript leans towards the modelling, but I would hope that a more thorough analyses of the fMRI data could further strengthen the authors' case.

**The reviewer raises an excellent point with additional analyses that might be fruitful and give a more concrete evidence to how each component in the model is related to the data.**

**The rationale to focus on the Switch condition was motivated by the Switch-related engagement of the MD thalamus in rodents (Rikyhe et al., 2018), where the MD was particularly important when animals switched their behavior. For example, MD inhibition before block change slowed down the animal’s switching behavior appropriately in the next context (Rikyhe et al., 2018). This pattern of impairment revealed that distinct machinery and interactions are needed in the brain to orchestrate these behavioral switches. Following the criticism also raised by the two other reviewers, we now detailed our approach to the fMRI results and clarified how we defined the regions of interest.**

**We now reference these rodent findings at the outset of the fMRI results section (Lines 190-193):**

***“To draw direct parallels between the neural model and human data, we returned to our previously collected dataset [32], and took a different analytical approach from the published work. Mice experiments revealed inhibition of pre-limbic prefrontal region impaired performance and inhibition of MD lead to specific impairment at times when task contingencies changed [21].”***

**The idea of mapping model outputs directly onto fMRI time series to truly track the underlying states is terrific. However, after carefully considering this, we realized that our setup might not support these analyses. We are concerned with specific discrete trials when subjects decide to switch their behavioral strategy. Despite the trials being the same pseudorandomized sequence, each participant made different behavioral decisions. The model behaves similarly to the overall average participant behavior, but there is not enough data to fit a model specifically to each individual to capture their specific decisions in response to a sequence of trial outcomes. In other words, for the same sequence of trials, our model predicts the same behavioral sequence plus some noise, but cannot predict what latent states each participant had in mind. Accordingly, relating the latent values predicted by the OFC model to OFC fMRI data will likely be mismatched, as the OFC model has no way of predicting when the particular subject switched their latents, and the OFC model will switch its latents at a generic moment dictated by the Bayesian model. As such, our only option to capture these moments of change in participants' brains is to directly correlate the fMRI with the individual’s specific behavioral output.**

Minor:

1) In the introduction, the authors sometimes switch between pulvinar and MD thalamus (e.g. line 71, 72) - would the authors see them as equivalent?

**We thank the reviewer for their careful comment. The two nuclei are of a different hierarchical order and we now remove these sentences from the introduction.**

2) The task task figure (1A) does not seem to show the non-predictive (p = 0.5) context? Further to this, what do the eight circles represent? In figure 1C, it is not quite clear to me what is shown with the dots. I think more description in the caption could help. Overall, while I found all other figures rather straightforward to read and clear, I was quite much struggling with this one (see also my questions about model architecture above).

**Figure 1 panels have been updated with improved design to illustrate the content in a clearer way. We now include the non-predictive context in the illustration. Regarding Fig 1C, the dots representing responses of the model on each trials and also the ground truth rewarded strategy. Those were now clearly marked and redesigned to highlight the differences, we switched to using bars in hope it will be clearer. The panel in question included here for reference:**

**We also updated the captions to fully describe these new bars (lines 125-128):**

***“C. Mean task performance of human participants (purple) and of the neural model (green) plotted over 10 blocks of association levels, to show qualitative similarity (middle). Grey bars reflect the correct rule that was rewarded on that trial and green bars show the model response (bottom).”***

3) It seems that some literature is not cited correctly. In the introduction (line 56/57), two studies (Takahashi et al., Schultz et al.) are cited in support of OFC-dlPFC interactions, but as far as I remember neither of these investigated this topic.

**We apologize for these suboptimal citations and have now cited a more appropriate study from Yael Niv’s lab, and an additional elaborate study of cognitive performance on many tasks and observing interactions between the two regions.**

Leong YC, Radulescu A, Daniel R, DeWoskin V, Niv Y. Dynamic Interaction between Reinforcement Learning and Attention in Multidimensional Environments. Neuron. 2017;93: 451–463. doi:10.1016/j.neuron.2016.12.040

Nejati V, Salehinejad MA, Nitsche MA. Interaction of the Left Dorsolateral Prefrontal Cortex (l-DLPFC) and Right Orbitofrontal Cortex (OFC) in Hot and Cold Executive Functions: Evidence from Transcranial Direct Current Stimulation (tDCS). Neuroscience. 2018;369: 109–123. doi:10.1016/j.neuroscience.2017.10.042

4) I was wondering what specific purpose the HGF was used for? It only briefly comes up, almost in passing on page 5. It is mentioned that prior beliefs about outcome probability were weaker on switch compared to stay trials - and then the authors move on. This seems unnecessary to me, but maybe I'm missing something, in which case I would ask the authors to elaborate.

**We thank the reviewer for raising this point. We used the HGF model to calculate the prior belief which was correlated to the fMRI activity in vmPFC. Since the HGF results are not in the focus of our story, we now refer the reader to the original citation and also to our recent publication for details if desired.**

5) For the model MD lesion studies, blocks with 0.9 and 0.1 match probability were used - unlike main experiment with 0.9, 0.7, 0.5. 0.3, 0.1 - why?

**Having multiple association levels helped us ensure that our model is reasonably fit to human behavior. However, in dissecting the effects of MD lesions, the 0.7 and 0.3 association levels produced similar dynamics to 0.9 and 0.1, while the 0.5 association level had all models guessing at random, as trial outcomes were unpredictable by design.**

**We now mention this explicitly in the text:**

**Lines 285-287:**

**“*We noticed similar patterns with other association levels (70%, 50%, 30%) and we only show alternating 90% and 10% blocks in following experiments for clarity.*”**
